# Disruption of Established Bacterial and Fungal Biofilms by a Blend of Enzymes and Botanical Extracts

**DOI:** 10.4014/jmb.2212.12010

**Published:** 2023-03-10

**Authors:** Gitte S. Jensen, Dina Cruickshank, Debby E. Hamilton

**Affiliations:** 1 NIS Labs, 1437 Esplanade, Klamath Falls, Oregon 97601, USA; 2 NIS Labs, 807 St Geory St. Port Dover, Ontario NO A INO, Canada; 3 Researched Nutritionals, Po Box 224 Los Olivos, California 93441, USA

**Keywords:** *Borrelia burgdorferi*, drug-resistance, persister cells, *Pseudomonas*, *Staphylococcus*

## Abstract

Microbial biofilms are resilient, immune-evasive, often antibiotic-resistant health challenges, and increasingly the target for research into novel therapeutic strategies. We evaluated the effects of a nutraceutical enzyme and botanical blend (NEBB) on established biofilm. Five microbial strains with known implications in chronic human illnesses were tested: *Candida albicans*, *Staphylococcus aureus*, *Staphylococcus simulans* (*coagulase-negative*, *penicillin-resistant*), *Borrelia burgdorferi*, and *Pseudomonas aeruginosa*. The strains were allowed to form biofilm in vitro. Biofilm cultures were treated with NEBB containing enzymes targeted at lipids, proteins, and sugars, also containing the mucolytic compound N-acetyl cysteine, along with antimicrobial extracts from cranberry, berberine, rosemary, and peppermint. The post-treatment biofilm mass was evaluated by crystal-violet staining, and metabolic activity was measured using the MTT assay. Average biofilm mass and metabolic activity for NEBB-treated biofilms were compared to the average of untreated control cultures. Treatment of established biofilm with NEBB resulted in biofilm-disruption, involving significant reductions in biofilm mass and metabolic activity for *Candida* and both *Staphylococcus* species. For *B. burgdorferi*, we observed reduced biofilm mass, but the remaining residual biofilm showed a mild increase in metabolic activity, suggesting a shift from metabolically quiescent, treatment-resistant persister forms of *B. burgdorferi* to a more active form, potentially more recognizable by the host immune system. For *P. aeruginosa*, low doses of NEBB significantly reduced biofilm mass and metabolic activity while higher doses of NEBB increased biofilm mass and metabolic activity. The results suggest that targeted nutraceutical support may help disrupt biofilm communities, offering new facets for integrative combinational treatment strategies.

## Introduction

A microbial biofilm is a community of adherent microbial cellular forms with properties that help protect the microbial community from disruption by physical, chemical, or immunological attack. Microbial forms living in biofilms are morphologically and functionally distinct from those of free-floating (planktonic) forms of the same species. Biofilms have greater resistance to chemical, physical, and immunological insults than the planktonic forms from the same species [1]. Microbial biofilms pose a major medical and industrial challenge due to resistance to chemical treatments, antibiotics, and an ability to evade immune recognition [2]. As a result, there is a strong research focus on identifying methods to discourage biofilm initiation and formation, and to disrupt existing biofilms [3].

Microbial biofilms form on liquid/solid interfaces in nature, such as rocks and clay particles and decaying plant materials. Biofilms also form on metals and plastics, including medical devices and implants, causing device-related infections, which are associated with a large majority of hospital-acquired infections [4-6]. Biofilms also form on body surfaces, such as the mucosal membranes in the gut, bladder, eye, ear, and lung, as well as in chronic wounds [2]. Persistent biofilm infections can induce a hyper-inflammatory state in the host [7], and include many chronic inflammatory infections including gastrointestinal tract [8], urinary tract, otitis media, infective endocarditis, cystic fibrosis [9], and dental plaque [10].

Biofilms secrete a complex mucus polymer structure that plays a role in microbial adhesion, cell-to-cell interactions, antimicrobial resistance, and immune evasion [11, 12]. The structural framework of the biofilm matrix contains many types of polymers, including polysaccharides, proteins, lipids, bacterial cellulose, and extracellular DNA that offers structural and functional protection [13]. The organisms living within the biofilm need to be able to communicate with each other in a process called quorum sensing [14]. Biofilms may contain multiple species coexisting and collaborating.

The difficulty in treating biofilm infections with pharmaceutical antibiotics and antimicrobials has led to a search for new treatment approaches, including disruption of the protective biofilm matrix, disruption of biofilm adhesion to the substrate, disruption of intra-biofilm communication via quorum sensing, and altering the gene expression of the microbe to be unable to sustain the biofilm environment (Table 1) [6]. When the microbes are no longer able to maintain the biofilm environment, they may revert to a free planktonic state that is more vulnerable to antimicrobial treatments and more visible to the immune system [15].

Enzymes that degrade biofilm polymers have been shown to inhibit new biofilm formation, detach existing biofilm colonies, and increase sensitivity of the biofilm to antimicrobial treatments [16], with the goal of reverting the microbial forms back to their planktonic state [6]. Combinations of antimicrobials that interfere with quorum sensing and adhesion with biofilm-disrupting agents offer additional strategies [17-21].

Based on the composition of biofilm matrices, enzymes are identified that can disrupt biofilm. Lysozyme is an enzyme that is naturally present in mucosal secretions and tissues of animals and humans as part of our innate immune system and able to disrupt bacterial biofilms [22, 23]. B-1,3-glucan is a vital component of fungal biofilms including *Candida* species, and glucanase enzymes can break down *Candida* biofilms and increase susceptibility to anti-fungals [24]. Enzyme cocktails including hemicellulases have been used on industrial scales to disrupt biofilms from biopolymer surfaces [25]. Lipase digests fats in the biofilm, making this enzyme important in breaking down both fungal and bacterial biofilms [26]. Many types of protease enzymes are helpful in breaking down the protein matrix and contribute to successful eradication of biofilms [27], leading to increased efficacy of antibiotics [28].

Biofilm formation requires a different gene expression profile than the free-floating microbial forms [29, 30]. Therefore, natural, or synthetic compounds that affect those aspects of microbial gene expression may discourage biofilm formation [31], and force microbes into the free-floating form that is more recognizable by the immune system. Examples include synthetic compounds [32], botanicals [33, 34], essential oils [35], secreted metabolites from beneficial probiotic bacteria [36], and bee venom [37].

Some herbs can inhibit the quorum sensing communication between microbes that contributes to the development of biofilms. Cranberry is well known for its use in preventing and treating urinary tract infections [38]. This is in part due to its ability to prevent and disassemble biofilms by multiple mechanisms including anti-adhesion, decreasing quorum sensing, and direct anti-microbial effects [39, 40]. Berberine, rosemary, and peppermint are other herbs that have been shown to be antimicrobial, anti-quorum sensing, and contributing to biofilm breakdown [41-43]. In addition to herbs, amino acids such as N-acetyl cysteine (NAC) are mucolytic and effective for eliminating bacterial biofilms [44].

We evaluated the biofilm-disrupting properties of a nutraceutical enzyme and botanical blend (NEBB) that contained a combination of enzymes and botanical antimicrobial extracts (Table 1). NEBB was tested for effects on disrupting established biofilms of five biofilm-forming microbial species, including one fungal species and four bacterial strains (Table 2). The purpose of this work was to conduct an initial screening for the effects of a consumable nutraceutical formulation, used by medical doctors to support the treatment of patients with severe chronic illnesses with suspected microbial biofilm involvement, which includes *Candida* and *Staphylococcus* subspecies. The types of patients who use this nutraceutical formulation under the supervision of doctors also include patients with chronic Lyme disease, *i.e.*, infection by *Borrelia burgdorferi*.

## Methods

### Reagents

Bacterial culture media were purchased from Sigma-Aldrich Inc (USA): Nutrient Broth (Catalogue number 70122), Tryptic Soy Broth (Catalogue number T8907), and BSK medium with 6% rabbit serum (Catalogue number B8291). The 96-well culture plates were obtained from Thermo-Fisher Scientific (USA): CellStar (Greiner Bio-One, Catalogue number 655-180) for all microbes except *Borrelia burgdorferi* for which collagen-coated 96-well plates were used (Catalogue number 152038). Other reagents were Crystal Violet (Catalogue number V5265, Sigma-Aldrich and CyQUANT VyBrant MTT cell viability assay kit (Catalogue number V13154, Invitrogen, Thermo-Fisher Scientific).

### Nutraceutical Enzyme and Botanical Blend

The nutraceutical enzyme and botanical blend (NEBB), BioDisrupt, was provided by the manufacturer, Researched Nutritionals, Los Olivos, CA, USA. The product is a powder that contains water-soluble enzymes (Table 1) and botanical extracts and N-acetyl Cysteine (Table 2).

In order to ensure the product was sterile, and would not introduce bacteria, yeast, or mold spores into the microbial cultures, the product was irradiated at 10 kGy. At NIS Labs, a sample of the powder was tested on Petrifilm culture plates to ensure there were no detectable aerobic bacteria, yeasts, or mold in the test product. Sterile emulsions were prepared from NEBB and introduced into the microbial biofilm cultures. Serial dilutions were tested across a broad dose range. Initial dose response testing revealed the ideal dose in biofilm cultures of this nutraceutical formulation, designed for human consumption, covered a range from 1 – 40 mg/ml, which is 10-fold higher than the dose range used for testing of antimicrobial effects of highly purified compounds.

### Microbial Strains and Culture Methods

Five microbes, known for their ability to live in biofilms, were included in this testing (Table 3). The 5 microbial strains – 1 fungal and 4 bacterial – were purchased from the American Type Culture Collection. The recommended culture media for each strain was used, and cultures were performed under conditions that encourage biofilm formation. The testing for effects of the nutraceutical blend on biofilm disruption involved these steps: 1) Culture each microorganism to facilitate biofilm formation in flat-bottom 96-well culture plates, 2) Add NEBB and continue culture for 24 h, 3) Remove planktonic (free) forms including disrupted biofilm and forms released from biofilm and wash the remaining biofilm with physiological saline, 4) Evaluate the estimated mass and metabolic activity of the remaining biofilm in untreated versus treated cultures.

### Removal of Planktonic Forms

The treatment of established biofilm with NEBB and the resulting disruption of biofilm included release of planktonic forms into the culture supernatant and detachment of clumps of bacteria living in biofilm. In order to provide conclusive data on the effects on biofilms, following published methodology [62], planktonic forms had to be removed from the cultures before staining for biofilm mass and metabolic activity, The removal of planktonic forms and the addition of washing buffer was done using a very low speed to avoid mechanical removal of biofilm material. The removal of planktonic forms was performed using electronic 12-channel pipettes (Viaflo, Integra, USA), where the speed was set to “1” (the maximum speed is “10”). Phosphate-buffered saline was added, also using speed “1”, where the liquid was dispensed onto the sidewalls of each well to avoid disruption of biofilm by direct pipetting actions onto biofilm. For the cultures of *Pseudomonas aeruginosa*, this pipetting allowed scoring of slime formation, where “0” indicated no change in viscosity of the culture medium, and a score of “3” (300%) indicated that the entire culture medium had turned into a mucus plug.

### Crystal Violet Staining for Biofilm Mass

The quantitative evaluation of biofilm mass for each microbial form was determined by crystal violet staining [63]. The saline was removed from each well and a 0.1% solution of Crystal Violet was added. The biofilm cultures were allowed to incubate with the crystal violet solution for a minimum of 10 min at room temperature, after which the culture plates were washed in distilled water. The distilled water was removed, and the stained biofilm plates allowed to air dry. The crystal violet was solubilized in 10% acetic acid for 15 min and the optical density measured by a plate-based spectrophotometer at 550 nanometers. The crystal violet staining was a measure for the relative mass of biofilm in each well. Untreated cultures served as a control for maximum biofilm formation. Blank wells without microbial forms served as negative controls. The percent-inhibition of biofilm formation was calculated for each dose of the test products.

### Biofilm Metabolic Activity Using the MTT Assay

A second set of culture plates was washed in the same way as the plates used for Crystal Violet staining. The culture plates were tested for metabolic activity using the MTT assay, which involves a colorimetric reaction based on cellular metabolic activity [64]. The MTT assay has been used for testing of metabolic activity in multiple types of biofilm [65, 66]. In this bioassay, chemical reactions involved in cellular metabolic reactions where oxidoreductase enzymes reduce the tetrazolium dye 3-(4,5-dimethylthiazol-2-yl)-2,5-diphenyltetrazolium bromide (MTT) to insoluble formazan crystals that are purple in color. The crystals are solubilized by addition of the detergent sodium dodecyl sulfate. The color development is measured by micro-plate-based spectrophotometry where the optical density is measured at 570 nanometers, using a PowerWave plate reader (BioTek Instruments, USA).

### Statistical Analysis

Average and standard deviation for each data set was calculated using Microsoft Excel. Statistical analysis was performed using the 2-tailed, independent t-test. Statistical significance was set at *p* < 0.05, and a high level of significance at *p* < 0.01.

## Results

### Disruption of Established Microbial Biofilm

The treatment of established microbial biofilms in vitro with NEBB showed reduced biofilm. The types of observations varied between the different microbial species.

**Fig. 1:**
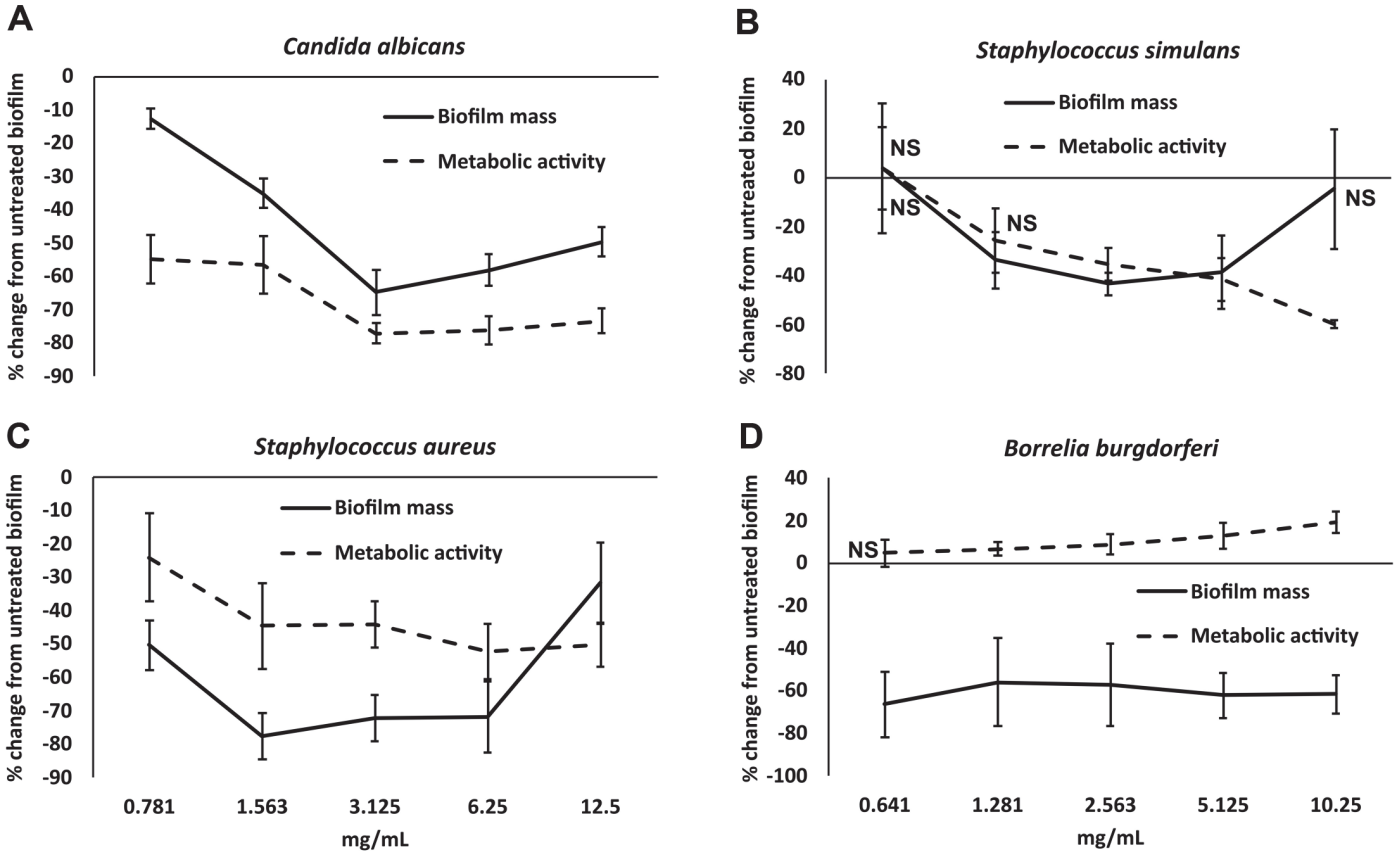
Disruption of established microbial biofilm from *Candida albicans* (A), *Staphylococcus simulans* (B), *Staphylococcus aureus* (C), and *Borrelia burgdorferi* (D) after treatment with a nutraceutical enzyme and botanical blend across a dose range of 0.8 – 12.5 mg/ml. Data is shown as the average + standard deviation of nine repeats of each treatment dose, as the % change from untreated biofilm, where the untreated biofilm had gone through identical procedures for removal of planktonic forms, washing, and addition of fresh medium. Biofilm mass was quantified by crystal violet staining (solid lines), and the metabolic activity of the microbial biofilms was measured by the MTT assay (dashed lines). All data points were statistically significant when compared to the untreated control biofilm cultures, except where the data point is annotated by NS (not significant).

*Candida albicans* in culture rapidly formed robust biofilms. NEBB was capable of disrupting these established *C. albicans* biofilm within 24 h (Fig. 1A). The relative mass of biofilm was significantly reduced at all doses of NEBB. At the dose of 3.125 mg/ml, the biofilm mass was 60% reduced compared to untreated cultures. This was also reflected in the reduced metabolic activity in proportion to the reduced biofilm mass. At the mid-dose, the relative metabolic activity of the cultures was 75% reduced compared to untreated control cultures. The effect of NEBB was highly significant across all doses of the product.

Established biofilm of the coagulase-negative, penicillin-resistant strain of *S. simulans* biofilm were also disrupted by NEBB at some doses. The relative mass of biofilm was significantly reduced at 1.281 – 5.125 mg/ml doses of NEBB. The dose response showed an unexpected increase in biofilm mass and metabolic activity at the lowest dose tested (Fig. 1B). It is possible that *S. simulans* sensed the effects of some ingredients even at this low dose and was able to respond by strengthening the biofilm as a protection.

The treatment of established biofilms of *S. aureus* resulted in rapid disruption of the biofilm, and this disruption was also associated with reduced metabolic activity (Fig. 1C). The reduced mass and metabolic activity of biofilm was significantly reduced at all doses of the product. For the reduced metabolic activity, the response showed a clear dose-dependent effect.

The effect of NEBB on mature biofilms from *B. burgdorferi* was more complex. The biofilms were established over a period of 5 weeks, leading to robust clusters of bacterial aggregates, where the morphology of the bacteria living in the biofilm showed dramatic changes from the free planktonic form of the spirochete. The biofilm formation and maturation were similar to published work from Sapi’s team [67], showing initial clustering of free planktonic forms, followed by disappearance of flagella, and increased encasement of the biofilm into hard structured aggregates adhering to the collagen-coated plastic surfaces. The clusters were connected by a dense network of spirochetal bridges, the relative metabolic activity was low, suggesting the bacteria adapted to the biofilm existence by converting into quiescent ‘persister’ cells [68]. When the *B. burgdorferi* biofilm was treated with NEBB for 24 h, there was a marked reduction in the biofilm mass, however, in contrast to the other bacterial forms, there was a mild increase in metabolic activity, suggesting that the bacterial forms returned into a more active state and were no longer as quiescent (Fig. 1D).

**Fig. 2:**
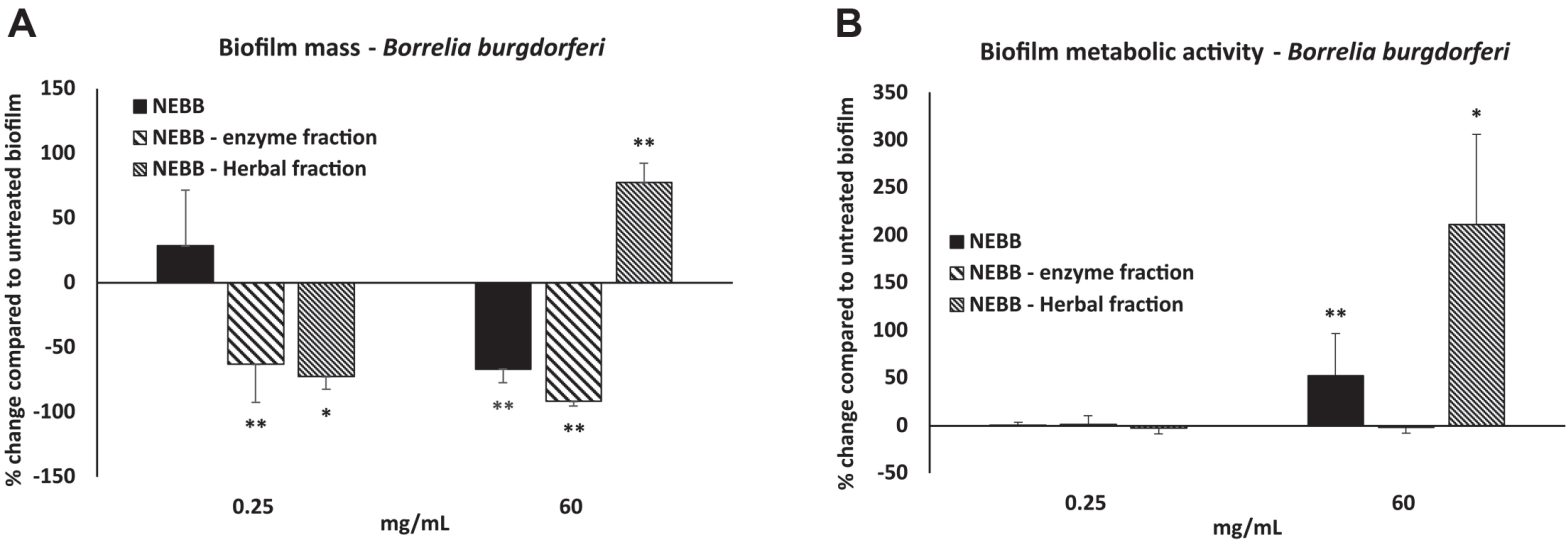
Effects on established bacterial biofilm from *Borrelia burgdorferi* on biofilm mass (A) and biofilm metabolic activity (B) after treatment with a nutraceutical enzyme and botanical blend (NEBB), compared to treatment with the NEBB enzyme fraction versus the NEBB herbal fraction. Data is shown as the average ± standard deviation of nine repeats of each treatment dose, as the % change from untreated biofilm, where the untreated biofilm had gone through identical procedures for removal of planktonic forms, washing, and addition of fresh medium. Levels of statistical significance are shown on the graphs where changes compared to untreated biofilm is indicated by asterisks, where *p* < 0.10: (*), *p* < 0.05: * and *p* < 0.01: **.

This prompted further testing of Borrelia biofilm cultures, to examine which fraction, the enzyme or the herbal, affected the metabolic activity the most (Fig. 2). Borrelia biofilm treated with the enzyme fraction showed reduced biomass (Fig. 2A) but the enzyme fraction had no effect on the metabolic activity (Fig. 2B). In contrast, the highest dose of the herbal fraction had a statistically significant increase in Borrelia biofilm metabolic activity (Fig. 2B).

Established biofilms of *P. aeruginosa* showed complex responses to treatment with NEBB. At the higher doses, NEBB reduced both the biofilm mass and metabolic activity at 30 mg/mL, and the disruption was highly significant (*p* < 0.01). However, at lower doses, NEBB triggered a defensive response in *P. aeruginosa* to strengthen the biofilm; this was seen both for the relative biofilm mass and for the metabolic activity (Fig. 2). There was approximately 600% increase in relative biofilm mass and 300% increase in metabolic activity. This increase was highly significant.

Another observation associated with NEBB treatment was slime production. *P. aeruginosa* is notorious for producing slime, and when growing in liquid suspensions in culture flasks, will display veils of slimy material. In 96-well microplates, the entire volume of the liquid cultures in each well can turn into slime plugs, making pipetting a challenge. We noticed that at the lower doses of NEBB the slime formation was increased compared to untreated cultures, and also compared to higher doses of NEBB. The observation suggests that while at higher doses NEBB mildly inhibited both biofilm and slime formation, at lower doses the microbe made massive biofilms to protect itself from NEBB. At the lowest dose, *P. aeruginosa* did not make biofilm but increased slime production as another evasive tactic.

## Discussion

The disruption of persistent immune-evasive and treatment-resistant microbial biofilms is a focus for research into novel types of treatments. Given today’s alarming problems with antibiotic-resistant bacteria, it is a desirable solution to evaluate non-pharmacological, non-antibiotic natural strategies [31].

Biofilms are the predominant form of existence of many microorganisms, including bacteria and simple fungi. The definition of biofilm involves a broad description of single-species, or multi-species, microbial community structures observed in both natural and laboratory environments. Treatment of microbial biofilms is recognized as an urgent need, in light of the multi-species communities inhabiting biofilm, and the resistance of such biofilms to conventional pharmaceutical treatments. Novel therapeutic strategies include combinations of enzymes targeted at the various matrix components surrounding adherent biofilm colonies, for a comprehensive approach to disrupting established biofilm [69]. Since many botanical compounds have also been associated with effects on biofilm survival and function, it was of interest to study the effect of a complex nutraceutical blend of enzymes and botanical extracts (NEBB), designed to break up established biofilm in the gut and tissue. NEBB was tested on established biofilms on 5 microbial species, selected based on their known ability to form biofilm, and the widely known association of these biofilms with chronic health problems.

We report here that established biofilms exposed to NEBB showed reduced biofilm mass when using crystal violet staining. The effects of NEBB on *C. albicans* and 2 species of *Staphylococcus* showed rapid disruption of biofilm, as seen by reduced biofilm mass; we suggest that the reduced metabolic activity in the cultures were in direct correlation to the reduced amount of biofilm. The reduction of *S. aureus* biofilm has multiple applications, since *S. aureus* biofilms are associated with multiple diseases, including sinus, ear, bone, heart, and non-healing wounds and infections in replacement joints. The reduction of *C. albicans* biofilm has direct implications for gut health since this microscopic yeast is known to be capable of forming biofilm along the intestinal mucosal barrier [70].

*Pseudomonas aeruginosa* is involved in severe acute and chronic infections, known to often involve other species such as commensal bacteria [71]. The effects of NEBB on *P. aeruginosa* involved a bi-phasic dose response where low doses of NEBB triggered significant reduction in biofilm, but higher doses triggered enhanced biofilm formation. This may possibly be due to evasive behavior by *Pseudomonas* exposed to high doses of NEBB. Further work should evaluate whether the biofilms showing enhanced biofilm formation were less virulent and inflammatory than untreated biofilms.

The spirochete *B. burgdorferi* is the causative agent of Lyme disease, a multisystemic disorder impacting primarily the skin, nervous system, and musculoskeletal functions, including Lyme arthritis. Immune cell-mediated clearance of *B. burgdorferi* infections depend in part on immune recognition and phagocytosis of free planktonic bacterial forms; this is hampered by bacterial biofilm formation [72]. We tested the effects of NEBB on *B. burgdorferi* and, in contrast to the other microbes tested, the reduction in biofilm was accompanied by an increase in metabolic activity. The observation that NEBB was able to change the metabolic state of *B. burgdorferi* and reduce the biofilm mass is clinically important. The stationary phase of persister cells with low metabolic activity [70] has been associated with more severe illness in a rodent model of Lyme arthritis [73], likely as a result of dysregulated hyper-inflammatory response that persists after the bacteria have either been cleared from the host [74], or taken on an obscure immune-resistant and antibiotic-resistant existence in various tissues in the form of quiescent biofilm [75].

The preliminary results reported here point to further directions for research, including biofilms associated with gut mucosa, as well as bacterial biofilms in tissue such as cartilage, and research involving intracellular biofilm-like colonies [76, 77]. This work, although novel and highly necessary, has limitations. Further research is needed involving multispecies biofilm, such as Borrelia/Candida or Borrelia/Staphylococcus co-cultures, and should include transcriptomics to evaluate changes to gene expression in co-cultures with or without treatment with NEBB, based on recent publications on multispecies biofilm [62, 78, 79]. In a clinical situation, biofilm will likely consist of multiple species, with an unknown combination of bacterial types, assisting the maintenance of the biofilm environment to protect itself from immune-mediated biofilm elimination. Our diagnostic methods are limited by tools available, and access to the deep tissue areas where biofilm may reside in quiescence avoiding detection [76].

We conclude that a targeted blend of botanical extracts and enzymes directed at biofilm matrix components is efficacious of disrupting established biofilm in vitro. This does not prove efficacy in a clinical situation. Further studies should establish whether NEBB disrupts for example *Candida* or *Staphylococcus* biofilm in the gut mucosa, as well as established biofilm in tissues. Work is in progress to evaluate the hyper-inflammatory effects of Borrelia biofilms and evaluate ways to reduce this inflammatory activity of the bacterial colonies (manuscript in preparation).

There is a great and urgent need for further research into complex biofilm communities. This is a well-known territory in geological sciences [80] but is in its infancy in medical science. Established biofilms may be more inflammatory and virulent than free planktonic forms, and further research should include proteomic evaluation of such stressors from complex multi-species biofilms.

## Figures and Tables

**Fig. 3:**
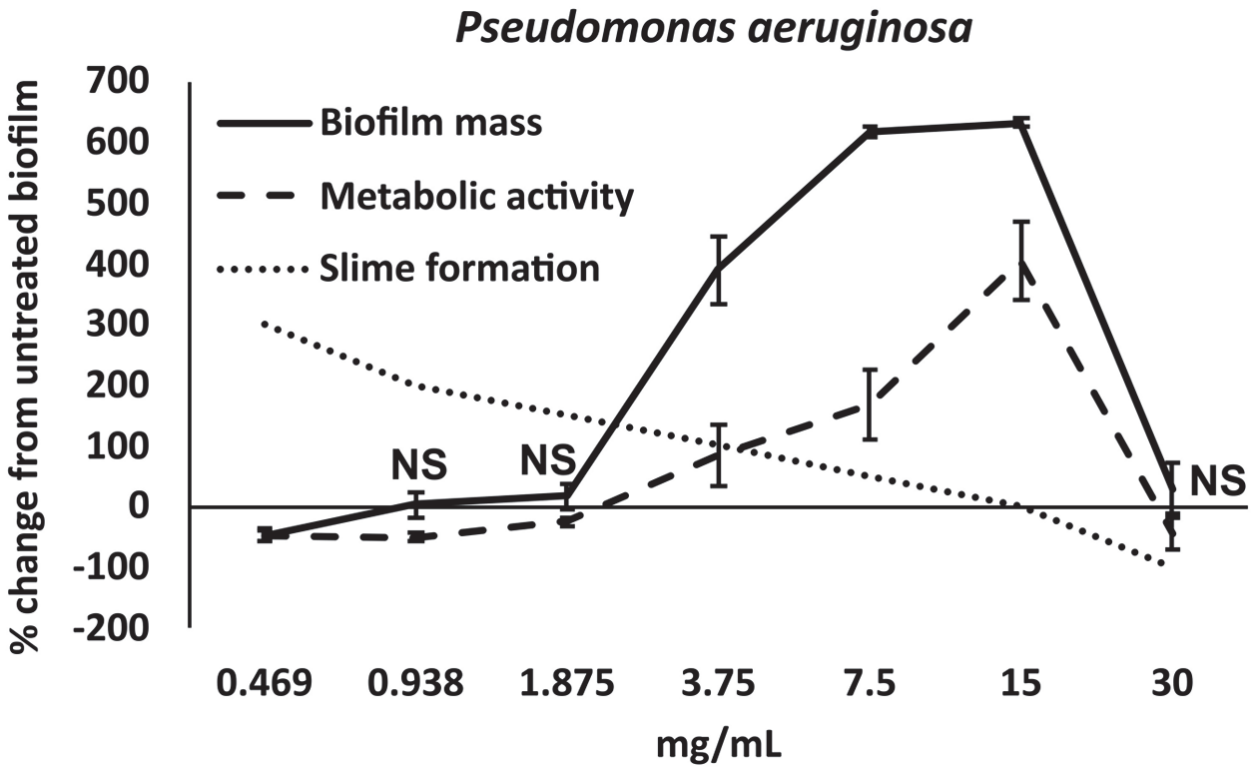
Disruption of established microbial biofilm from *Pseudomonas aeruginosa* after treatment with a nutraceutical enzyme and botanical blend across a dose range of 0.5 – 30 mg/ml. Data is shown as the average ± standard deviation of a minimum of 3 repeats of each treatment dose, as the % change from untreated biofilm, where the untreated biofilm had gone through identical procedures for removal of planktonic forms, washing, and addition of fresh medium. Biofilm mass was quantified by crystal violet staining (solid lines), and the metabolic activity of the microbial biofilms was measured by the MTT assay (dashed lines). Slime formation was scored with “3” (300%) change indicating that each culture in the microtiter plate had turned to a mucus plug. All data points were statistically significant when compared to the untreated control biofilm cultures, except where the data point is annotated by NS (not significant).

**Table 1:** Enzymes in the nutraceutical enzyme and botanical blend.

Enzymes– 67 mg/oral dose	Target specificity	Disruption of biofilm
Lysosyme (from hen’s egg white)	Peptidoglycans in bacterial cell walls	[45, 46]
Serratiopeptidase	Proteins	[47, 48]
Beta-glucanase	Carbohydrates including fungal beta-glucans	[49, 50]
Lipase	Lipids	[26, 51]
Protease	Proteins	[27, 28]
Cellulase/ Hemicellulase	Bacterial cellulose	[52, 53]

**Table 2:** Herbal ingredients in the nutraceutical enzyme and botanical blend.

Herbal Ingredients – 905 mg/oral dose*	Types of anti-microbial effects
Cranberry (fruit) extract	Inhibition of bacterial biofilm formation [38, 39], inhibition of quorum-sensing [40]
Berberine	Growth inhibition [41, 54], inhibition of bacterial [55] and fungal [56] biofilm formation
Rosemary (leaf) extract	Inhibition of bacterial [57] and fungal [58] biofilm formation
Peppermint oil powder	Inhibition of bacterial biofilm formation [59], disruption of quorum sensing [60]
N-acetyl cysteine	Growth inhibition [44], Disrupts mucins [61]

*This includes 300 mg of N-acetyl cysteine

**Table 3:** Microbial strains and culture media.

Microbial strain	Culture medium (Duration, temperature)
*Candida albicans* (Robin) Berkhout (ATCC 10231)	Yeast malt broth (24 h, 37°C)
*Staphylococcus aureus* subsp. aureus Rosenbach (ATCC 6538)	Tryptic soy broth (24 h, 37°C)
*Staphylococcus simulans* Kloos and Schleifer (ATCC 11631)*	Nutrient broth (24 h, 37°C)
*Pseudomonas aeruginosa* (Schroeter) Migula (ATCC 9027)	Nutrient broth (24 h, 37°C)
*Borrelia burgdorferi* Strain B31 (ATCC 35210)	BSK-H complete medium (35 days, 33°C)

*Coagulase-negative, penicillin-resistant.
